# Unexpected sudden death on arrival in a healthy middle-aged man associated with COVID-19-related diffuse cardiac injury: A case report

**DOI:** 10.1016/j.heliyon.2023.e23460

**Published:** 2023-12-10

**Authors:** Shotaro Isozaki, Yu Kakimoto, Haruka Ikeda, Yutaka Matsushima, Akio Tsuboi, Motoki Osawa

**Affiliations:** Department of Forensic Medicine, Tokai University School of Medicine, Isehara 259-1193, Japan

**Keywords:** SARS-CoV-2, Myocardial injury, Platelet aggregation, Sudden Unexpected death

## Abstract

Coronavirus disease 2019 (COVID-19) is an emerging respiratory infectious disease caused by severe acute respiratory syndrome coronavirus 2 (SARS-CoV-2). COVID-19 has been reported to lead to acute cardiac injury, but previous research indicated that the mechanism is different from that of other viruses and remains poorly understood. Herein, we describe a case of COVID-19-associated sudden death, in a healthy 47-year-old man after developing diffuse cardiac necrosis. Two days before death, the patient developed general malaise without respiratory symptoms. The patient's fatigue worsened with time, and he ultimately developed cardiac arrest in an ambulance; however, resuscitation was unsuccessful. Antigen testing performed at the hospital revealed that the patient was positive for SARS-CoV-2 virus. At autopsy, contraction band necrosis was observed insularly in all areas of the myocardium. CD42b immunohistochemical staining indicated platelet aggregation in the microvessels around the cardiac necrosis area, suggesting COVID-19 can be fatal for healthy people by microcirculatory disturbance due to diffuse cardiac injury arising from platelet activiation. This unique mechanism can be a novel therapeutic target of COVID-19-related cardiac injury.

## 1 Introduction

Coronavirus disease 2019 (COVID-19) is a newly emerging infectious disease caused by the severe acute respiratory syndrome coronavirus 2 (SARS-CoV-2) virus. SARS-CoV-2 causes respiratory injury including pneumonitis, bronchitis, and pharyngitis [1]. COVID-19 is also associated with a severe and fatal course, which does not often occur in healthy middle-aged adults but rather in old-aged adults or those who have underlying health conditions such as hypertension, diabetes, and severe obesity [[1], [2], [3]]. In Japan, the mortality rate due to COVID-19 is less than 0.5 % as of 2022 (https://covid19.mhlw.go.jp/extensions/public/en/index.html).

In most fatal cases, direct cause of death due to SARS-CoV-2 infection is pulmonary system dysfunction such as pneumonia and bronchitis [4]. SARS-CoV-2 also causes life-threatening cardiac injury including myocarditis. Previous clinical research based on cardiac magnetic resonance imaging and myocardial markers showed that more than 20 % of COVID-19 patients developed myocardial injury [5,6]. However, another report based on autopsy findings concluded that only 1.4 % of autopsy cases showed typical myocarditis histology [7]. These contrasting findings indicate that SARS-CoV-2 may have a unique mechanism of causing cardiac injury, which is different from that of acute myocarditis caused by other viruses such as parvovirus, human herpes virus, and coxsackievirus. However, the mechanism and histology have yet to be fully understood.

We report an autopsy case of SARS-CoV-2-associated sudden death in a healthy 47-year-old man with diffuse cardiac necrosis and platelet aggregation in cardiac microvessels.

## 2 Case

The deceased was a 47-year-old man who lived with his family with an uneventful medical history. The patient was not vaccinated against the SARS-CoV-2 virus. Twelve days before the patient's death, his father developed a fever and was medically diagnosed as having COVID-19. Two days before the patient's death, he developed general malaise but did not complain of having a fever or any respiratory symptoms such as a cough or breathing difficulty. The patient's fatigue worsened with time, and he was unable to eat any meals the day before his death. Two hours before the patient's death, his mother called for emergency medical services. The patient presented with an ST-segment elevation on electrocardiogram and cardiopulmonary arrest occurred on the way to the hospital; he was pronounced dead after a failed resuscitation attempt without return of spontaneous circulation (ROSC). Laboratory tests performed at the hospital detected the SARS-CoV-2 antigen on an oropharyngeal swab with a concentration of more than 5000 pg/mL. Laboratory tests also revealed elevated cardiac marker levels including troponin and brain natriuretic peptide (BNP) and thrombopenia (Table 1). Three days after the patient's death, a complete autopsy, excluding craniotomy, was performed at our facility under written consent from a parent of the deceased and in compliance with the law of dissection and preservation of corpses. We also obtained informed consent from his parents for publication of the present case and its accompanying images before the autopsy.

**Table 1:** Patient's laboratory findings.

		normal range	Samples collected from
WBC ( × 10^3/μL)	12.8	3.3–8.6	emergency hospital
RBC ( × 10^6/μL)	5.91	4.35–5.55	emergency hospital
Hb(g/dL)	17.9	13.7–16.8	emergency hospital
Plt ( × 10^4/μL)	12.1	15.8–34.8	emergency hospital
CK-MB (U/L)	200	<25	emergency hospital
Troponin (pg/mL)	5980	<14	emergency hospital
BNP (pg/mL)	1732	<18.4	emergency hospital
CRP (mg/dL)	0.62	<0.14	emergency hospital
IL-1β(pg/mL)	164	<10	postmorterm blood
IL-6 (pg/mL)	2840	<4.0	postmorterm blood
IL-10 (pg/mL)	<2	<5	postmorterm blood
TNF-α (pg/mL)	0.41	<1.66	postmorterm blood

**Fig. 1:**
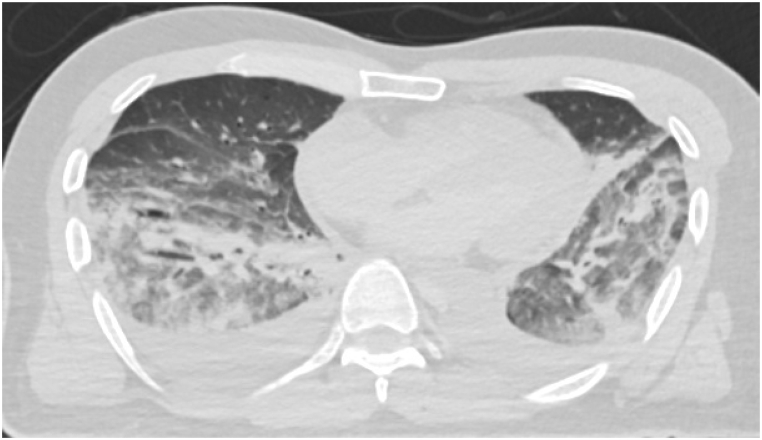
Postmortem computed tomography images. Axial image of the chest. Dorsal pulmonary infiltrative shadow and bilateral pleural effusions were detected. Cardiothoracic ratio was 55 %.

An unenhanced computed tomography scan was performed in the supine position prior to autopsy and revealed a dorsal pulmonary infiltrative shadow and bilateral pleural effusions; cardiothoracic ratio was 55 % (Fig. 1). We did not detect any lesions in the skull or abdominal cavity.

**Fig. 2:**
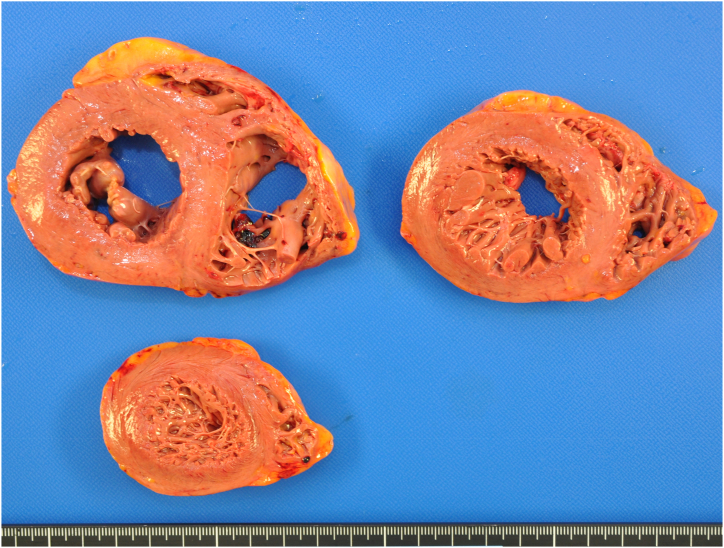
Macroscopic images of the heart. There were no macroscopic hemorrhage, fibrosis, and ischemic regions at the cardiac muscle surface.

The cadaver was 179.5 cm in height and 78.2 kg in weight (body mass index = 24.3 kg/m^2^) at the time of autopsy. Internal inspection revealed a pericardial effusion of 70 mL clear fluid. The heart weighed 426 g (normal range: 233–383 g [8]) and coronary arteries did not show arterial sclerosis or stenosis. Gross pathological changes were undetected in the heart muscles while performing the autopsy (Fig. 2). Left and right pleural effusions were 300 mL and 250 mL, respectively, and left and right lungs weighed 840 g and 1091 g (normal range: 122–675 g (left) and 155–720 g (right) [9]), respectively. The remainder of the macroscopic examination was unremarkable.

**Fig. 3:**
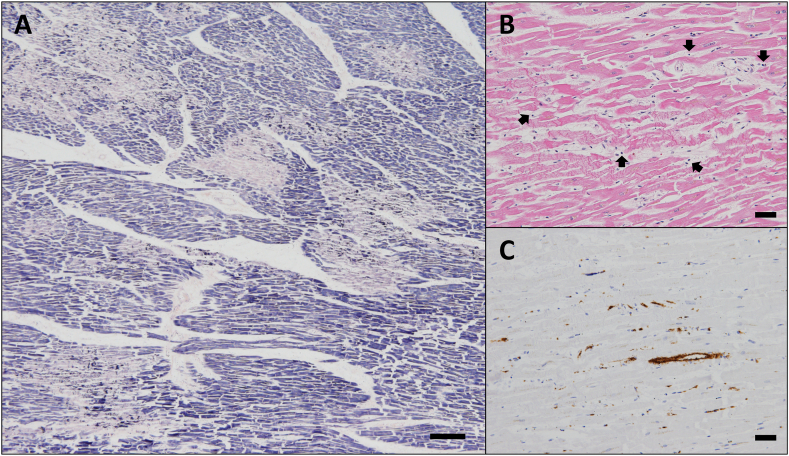
Heart histopathology findings. A. Patchy necrotic areas are diffusely scattered, and the striated pattern of cardiomyocytes is lost. Fibrin thrombus in the microvessels is not observed. Phosphotungstic acid hematoxylin (PTAH) stain. Scale bar: 200 μm. B. Contraction band necrosis is accompanied by several lymphocytes. H&E stain. Scale bar: 50 μm. The lymphocytes are indicated by black arrows. C. Platelets adhere to the endothelium and aggregate in the microvessels around the necrotic areas. Anti-CD42b stain. Scale bar: 50 μm.

**Fig. 4:**
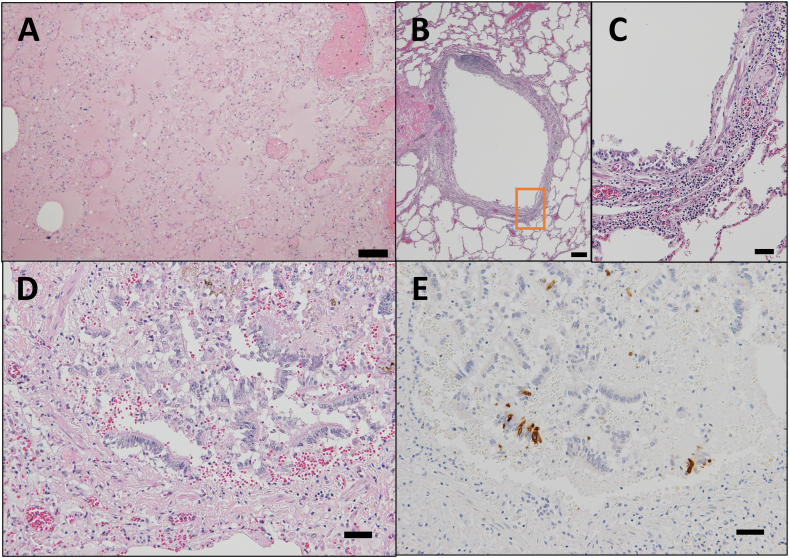
Lung histopathology findings. A. Pulmonary edema is widespread with a small number of macrophages without neutrophils. Scale bar: 100 μm. B, C. Mild level of lymphocytic infiltration is observed in some bronchioles. H&E stain. Scale bar: 200 μm (B) and 50 μm (C). D, E. SARS-CoV-2 positive cells were only found in the epithelial cells peeled off bronchioles. H&E and Anti-SARS-CoV-2 nucleocapsid stain. Scale bar: 50 μm.

Histopathological analysis showed diffuse contraction band necrosis with several lymphocytes in the cardiac muscle, despite no coronary stenoses (Fig. 3A–B). Platelet aggregation was detected in the microvessels around the myocardial necrosis by CD42b immunohistochemical staining (Fig. 3C), whereas there were no fibrin clot formations in the cardiac vessels. Moderate bronchiole inflammation and pulmonary edema with mild macrophage infiltration were detected, but there was no severe pneumonitis at the lung tissue (Fig. 4A–C). Fibrin clot formation was undetected in all organs including pulmonary artery. Immunohistochemical staining for SARS-CoV-2 nucleocapsid was selectively positive in some epithelial cells peeled off the bronchioles (Fig. 4D–E) and negative in the other organs including the heart. Postmortem blood tests revealed elevated inflammatory cytokines including interleukin −1 beta (IL-1β) and Interleukin-6 (IL-6) (Table 1). On the other hand, Interleukin-6 (IL-6) immunohistochemical staining showed there were no IL-6 positive cardiomyocytes in the myocardial necrosis area (Fig. S1). The result of the polymerase chain reaction test using samples from the pericardial effusion showed positive for SARS-CoV-2. Thus, we diagnosed the cause of death as COVID-19-related cardiac injury based on the autopsy findings and case history.

## 3 Discussion

This is a case of an otherwise healthy man who died of cardiac complications associated with SARS-CoV-2 infection. Severe COVID-19 patients frequently develop high fever and pulmonary symptoms including tussis and dyspnea [10]. However, in this case, he developed more general malaise rather than specific respiratory manifestations. We observed ST elevation on electrocardiogram and elevated levels of myocardial markers. A previous study based on forensic autopsy showed that myocardial marker levels, including troponin and BNP, affected neither the resuscitation attempt nor post-mortem interval [11]. Therefore, we diagnosed that he developed severe cardiac dysfunction prior to resuscitation. Autopsy findings showed no massive neutrophil invasion, pulmonary hyaline membrane formation, or diffuse alveolar damage in the patient's lungs, which indicated that he did not develop severe acute lung inflammation. In contrast, our autopsy findings revealed massive contraction band necrosis in cardiac muscles. Contraction band can be produced by ischemia-reperfusion injury including cases of ROSC following resuscitation [12,13]. In the present case, however, clinical history, electrocardiogram alteration, and elevated myocardial marker levels indicated he developed massive cardiac injury before resuscitation. Therefore, we determined cause of death to be myocardial injury induced by COVID-19. The finding of severe pulmonary edema in the lungs with increasing lung weights was believed to be caused by heart failure with high hydrostatic pressure, which was probably due to SARS-CoV-2 infection.

Myocardial injury due to viral infection is associated with acute heart failure and, although relatively uncommon, can cause sudden death. Typically, histopathological images of acute myocarditis caused by viral infections including enteroviruses, adenoviruses, parvovirus B19, and human herpesvirus 6 infections are characterized by myocardial necrosis with severe inflammatory cell infiltration [14]. However, a literature review of 277 autopsied hearts across 22 independent publications on COVID-19-positive patients identified that typical myocarditis histology including nonspecific inflammatory infiltrates was only seen in 4 cases (1.4 %) [7]. Moreover, this report also showed that focal necrosis frequency, described as “myocyte damage,” “scattered individual cell myocyte necrosis,” and “single cell ischemia,” was seen in 38 cases (13.7 %). Thus, this report suggests that SARS-CoV-2 presents a unique mechanism of cardiac injury preceding myocardial necrosis aside from acute inflammatory cell infiltration. In our case, the patient's cardiac muscle injury was also different from typical myocarditis due to widely insular contraction band necrosis with several lymphocytes. Immunohistopathological analysis showed no SARS-CoV-2 antigen-positive cells in cardiac muscles and platelet aggregation was located in the microvessels around the myocardial necrosis. We used CD42b as a platelet marker, which is a type I transmembrane protein that exists on platelet surfaces and is associated with binding von Willebrand factor [[15], [16], [17]]. A previous report using autopsy findings of people who died from COVID-19-associated pneumonia found contract band necrosis without coronary artery stenosis, absence of SARS-CoV-2 positive cells, and presence of platelet aggregation in cardiac microvessels [18]. The authors of this previous study regarded these findings as secondary changes due to severe respiratory dysfunction-related tissue hypoxia. However, in this present case, we detected CD42b-positive aggregated platelets at cardiac microvessels in the absence of pneumonia, indicating platelet aggregation of cardiac microvessels was not necessarily required severe pneumonia an it could be caused by primary platelet activation by SARS-CoV-2. Other previous reports also have indicated that SARS-CoV-2 causes a hypercoagulable state and platelet activation following binding CD42b existing surface of platelets and spike protein of SARS-CoV-2 [19,20]. In our case, we detected elevation of cytokines, including IL-1β and IL-6, indicating that the patient developed a cytokine storm. In contrast, we did not detect IL-6 positive cardiac myocytes around necrosis area. It is well researched that IL-1βand IL-6 activate platelets and cause aggregation [21,22]. Previous reports have showed that SARS-CoV-2 infection leads to the development of disseminated intravascular coagulation (DIC) and thrombocytopenia by multiple microvascular thrombosis following cytokine storm; DIC incidence has been reported as 0.6 % for survivors and 71.4 % for non-survivors, indicating that most fatal cases develop into DIC [23,24]. In view of previous studies, the cardiac necrosis in this patient developed not from direct necrosis due to cytokine including IL-6 but from microvascular thrombosis due to platelet aggregations and DIC. This case may present a characteristic mechanism of SARS-CoV-2-associated diffuse myocardial injury development via microcirculatory disturbance following platelet activation. Another previous report showed 11 out of 40 COVID-19 autopsy cases developed focal necrosis, while eight cases also developed micro-thromboembolism; however, this research is limited to only elderly adults (range of age: 65–81 years old) [25]. In view of several previous observational studies, indicating antiplatelet therapy to COVID-19 was associated with lower inpatient mortality [[26], [27], [28]], the present case claims the importance of platelet-targeted therapy for prevention of COVID-19-related sudden cardiac death for individuals in their 40s and for those even younger.

A limitation of this case report was that we were unable to perform a detailed assessment of the coagulating system due to lack of laboratory data, including data on protein C, protein S, antithrombin III, and D-dimer levels.

## Data availability statement

The data used to support the findings of this study are available from the corresponding author upon request.

## CRediT authorship contribution statement

**Shotaro Isozaki:** Writing – original draft, Resources, Investigation, Data curation, Conceptualization. **Yu Kakimoto:** Writing – review & editing, Resources, Data curation. **Haruka Ikeda:** Resources, Investigation. **Yutaka Matsushima:** Resources, Investigation. **Akio Tsuboi:** Resources, Investigation. **Motoki Osawa:** Supervision, Resources.

## Declaration of competing interest

The authors declare that they have no known competing financial interests or personal relationships that could have appeared to influence the work reported in this paper.
