# Supplementary figures and images for: Unexpected sudden death on arrival in a healthy middle-aged man associated with COVID-19-related diffuse cardiac injury: A case report
Source: Heliyon. 2023 Dec 10;10(1):e23460. doi: 10.1016/j.heliyon.2023.e23460 (PMC10758818; doi:10.1016/j.heliyon.2023.e23460)

## Slide 1
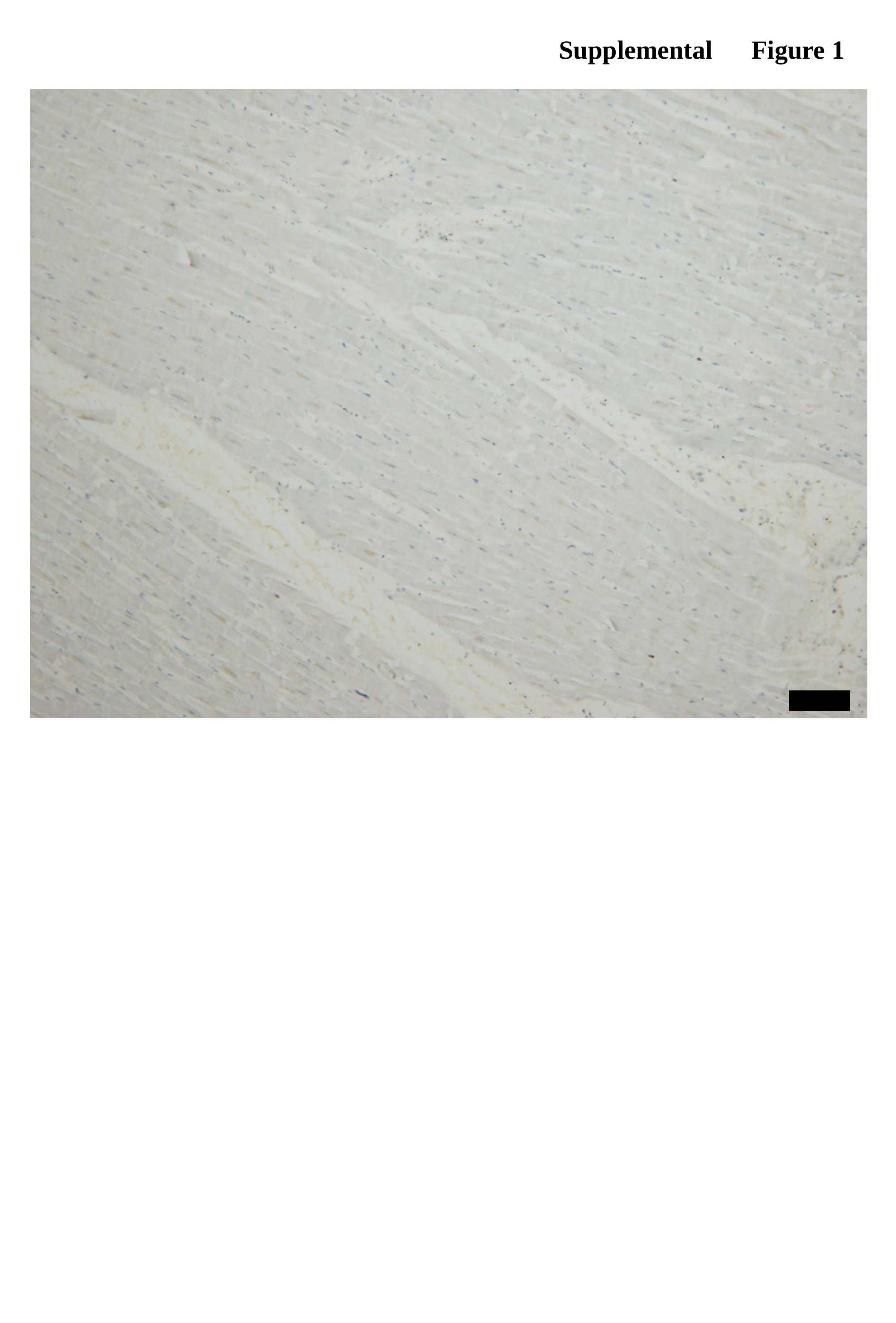

Supplemental　Figure 1

Supplement: Multimedia component 1 [file mmc1.pptx]
